# Functional genomic analyses of *Enterobacter*, *Anopheles* and *Plasmodium* reciprocal interactions that impact vector competence

**DOI:** 10.1186/s12936-016-1468-2

**Published:** 2016-08-22

**Authors:** Nathan J. Dennison, Raúl G. Saraiva, Chris M. Cirimotich, Godfree Mlambo, Emmanuel F. Mongodin, George Dimopoulos

**Affiliations:** 1 W. Harry Feinstone Department of Molecular Microbiology and Immunology, Bloomberg School of Public Health, Johns Hopkins University, Baltimore, MD USA; 2 Institute for Genome Sciences, University of Maryland School of Medicine, Baltimore, MD USA

**Keywords:** Mosquito, Malaria, Microbiota, Transmission-blocking, Oxidative stress, Sugar-bait

## Abstract

**Background:**

Malaria exerts a tremendous socioeconomic impact worldwide despite current control efforts, and novel disease transmission-blocking strategies are urgently needed. The *Enterobacter* bacterium *Esp_Z*, which is naturally harboured in the mosquito midgut, can inhibit the development of *Plasmodium* parasites prior to their invasion of the midgut epithelium through a mechanism that involves oxidative stress. Here, a multifaceted approach is used to study the tripartite interactions between the mosquito, *Esp_Z* and *Plasmodium,* towards addressing the feasibility of using sugar-baited exposure of mosquitoes to the *Esp_Z* bacterium for interruption of malaria transmission.

**Methods:**

The ability of *Esp_Z* to colonize *Anopheles gambiae* midguts harbouring microbiota derived from wild mosquitoes was determined by qPCR. Upon introduction of *Esp_Z* via nectar feeding, the permissiveness of colonized mosquitoes to *Plasmodium falciparum* infection was determined, as well as the impact of *Esp_Z* on mosquito fitness parameters, such as longevity, number of eggs laid and number of larvae hatched. The genome of *Esp_Z* was sequenced, and transcriptome analyses were performed to identify bacterial genes that are important for colonization of the mosquito midgut, as well as for ROS-production. A gene expression analysis of members of the oxidative defence pathway of *Plasmodium berghei* was also conducted to assess the parasite’s oxidative defence response to *Esp_Z* exposure.

**Results:**

*Esp_Z* persisted for up to 4 days in the *An. gambiae* midgut after introduction via nectar feeding, and was able to significantly inhibit *Plasmodium* sporogonic development. Introduction of this bacterium did not adversely affect mosquito fitness. Candidate genes involved in the selection of a better fit *Esp_Z* to the mosquito midgut environment and in its ability to condition oxidative status of its surroundings were identified, and parasite expression data indicated that *Esp_Z* is able to induce a partial and temporary shutdown of the ookinetes antioxidant response.

**Conclusions:**

*Esp_Z* is capable of inhibiting sporogonic development of *Plasmodium* in the presence of the mosquito’s native microbiota without affecting mosquito fitness. Several candidate bacterial genes are likely mediating midgut colonization and ROS production, and inhibition of *Plasmodium* development appears to involve a shutdown of the parasite’s oxidative defence system. A better understanding of the complex reciprocal tripartite interactions can facilitate the development and optimization of an *Esp_Z*-based malaria control strategy.

**Electronic supplementary material:**

The online version of this article (doi:10.1186/s12936-016-1468-2) contains supplementary material, which is available to authorized users.

## Background

The impact of malaria transmission upon human health cannot be overstated, with up to two billion people worldwide at risk and an estimated 438,000 deaths yearly [1]. *Plasmodium* parasites, the causative agents of malaria, are transmitted through blood-feeding female anopheline mosquitoes. *Plasmodium*’s complex life cycle provides multiple opportunities for intervention approaches, by targeting either the parasite or the mosquito vector. Strategies that target the parasite, including anti-malarial drugs, and those that target the vector, such as insecticides, are encountering resistance of the parasite and vector, respectively [2, 3]. When mosquitoes bite a *Plasmodium*-infected person, the ingested *Plasmodium* gametocytes encounter a severe bottleneck in development at the ookinete stage within the midgut, the site where most parasites are killed [4, 5]. During this infection stage, parasites also encounter the mosquito midgut microbiota. The negative impact of the mosquito microbiota on *Plasmodium* parasites has been well documented (reviewed in [6]) and offers an attractive opportunity to further narrow the bottleneck around *Plasmodium* development and its transmission between humans. The potential impact of the microbiota upon *Plasmodium* development suggests that studying these microorganisms could improve efforts at curtailing malaria transmission.

Interest in the tripartite interaction between vector, parasite, and microbiota has prompted the surveying of natural mosquito populations, revealing a microflora predominantly composed of Gram-negative species, with members of the *Proteobacteria* class often in high abundance [7, 8]. A number of naturally occurring bacteria have been shown to inhibit *Plasmodium* development in the mosquito, either through direct inhibition or indirectly through priming of basal immunity [9–11]. A correlation between *P. falciparum* infection and the prevalence of *Enterobacteriacae* has been documented [7], and several Gram-negative bacteria species have been shown to reduce the mosquitoes’ vectorial capacity [12–14]; also, a *Chromobacterium* species has been shown to possess anti-*Plasmodium* effects both in vivo and in vitro [15].

Screening isolates of the midgut microbiota of a Zambian mosquito population for *Plasmodium*-blocking activity has allowed the identification of an *Enterobacter* bacterium, *Esp_Z*, which is naturally harboured in the mosquito midgut tissue and can inhibit the development of *Plasmodium* parasites prior to their invasion of the mosquito midgut epithelium, independent of the mosquito’s immune system [10]. *Esp_Z* nearly eliminates the development of malaria parasites to the human-infective sporozoite stage, thereby showing potential as a transmission-blocking agent.

*Esp_Z,* even at low concentrations, is effective at inhibiting parasites in the mosquito intestine through a mechanism involving reactive oxygen species (ROS) [10]. *Plasmodium* parasites are susceptible to changes in the redox balance of their environment, and they defend themselves against ROS via an upregulation of antioxidant genes [16]. Two antioxidant systems with partially overlapping functions have been described in *Plasmodium* parasites, the thioredoxin and glutathione systems (reviewed in [17]). These systems are required to neutralize the highly oxidative environments encountered in both human erythrocytes [17] and the mosquito midgut [18]. The thioredoxin system is believed to be the main antioxidant system, since *P.falciparum* parasites lack a glutathione-dependent peroxidase [19, 20], suggesting that thioredoxin reduction of peroxiredoxin antioxidant enzymes plays a prominent role in protecting *Plasmodium* from toxic ROS [16]. Peroxiredoxin antioxidant enzymes such as peroxiredoxin-1 (Tpx-1) and 1-cys-peroxiredoxin (1-Cys-prx) act as electron acceptors from thioredoxin-1 (Trx-1), maintaining them in a reduced state [21]. *Plasmodium falciparum* Tpx-1 knockout lines show an increased sensitivity to both ROS and reactive nitrogen species, but the deletion mutation is not lethal, suggesting redundancy in the parasite’s antioxidant system [22]. A glutathione peroxidase-like thioredoxin peroxidase (TPx_Gl_) has also been shown to be part of the thioredoxin system in *Plasmodium* and is reduced by Trx-1 [23]. Inhibitors of the thioredoxin pathway have been investigated as potential anti-malarial compounds, rendering the parasite more susceptible to oxidative stress [24].

Recently, reintroduction of several bacteria through the nectar meal, a natural route and vital mosquito energy source, has been demonstrated to inhibit *Plasmodium* sporogonic development, and methods for exposing mosquitoes to various agents through artificial nectars have been developed [9, 15, 25]. Manipulation of the composition of the mosquito microbiota may offer a strategy to reduce the prevalence of *Plasmodium*-infected mosquitoes and, therefore, malaria transmission. A multifaceted approach was used to test the feasibility of using sugar-baited exposure of mosquitoes to the *Esp_Z* bacterium for interruption of malaria transmission, by studying the reciprocal interactions between the bacterium and the mosquito and its microbiota, together with *Esp_Z*’s impact on the parasite under laboratory conditions. The *Esp_Z* genes that are likely to be conducive to effective colonization of the mosquito midgut and ROS–mediated parasite inhibition were specifically assessed, along with the effect of *Esp_Z* exposure on the parasite’s oxidative defence system, mosquito fitness, and the ability of *Esp_Z* to compete with the endogenous bacteria of the mosquito midgut microbiota.

## Methods

### Mosquito rearing and antibiotic treatment

*Anopheles gambiae* Keele strain mosquitoes were maintained under laboratory conditions at 27 °C and 80 % humidity with a 14 h day/10 h night cycle. Larvae were reared on cat food pellets and ground fish food supplement. Adult mosquitoes were maintained on 10 % sucrose and fed on mouse blood (mice were anesthetized with ketamine) for egg production. For clearance of the endogenous bacteria of laboratory mosquitoes, adult female mosquitoes were maintained on antibiotics immediately after eclosion (75 μg/mL gentamicin sulfate [Quality Biological, Gaithersburg, MD, USA] and 100 μg/mL penicillin–streptomycin [Invitrogen, Carlsbad, CA, USA] in a 10 % sucrose solution ad libitum for three to 4 days). The sucrose-antibiotic solution was changed every 48 h. To minimize the impact of any residual antibiotics, the mosquitoes were allowed to feed ad libitum for 24 h on a sterile 10 % sucrose solution following antibiotic treatment.

### Bacteria cocktail preparation

The bacterial cocktail was prepared from bacterial isolates previously identified from wild *An. arabiensis* mosquitoes obtained through landing catches in Zambia [10]. Twelve species representing both Gram-negative and Gram-positive bacteria were selected. Single bacterial colonies from LB plates were selected and used to inoculate overnight cultures, then used to seed fresh cultures and grown to an OD_600_ of 1.0. Species names and GenBank sequence accession numbers were as follows: *Knoellia* sp. JF690939.1; *Acinetobacter* sp. JF690925.1; *Bacillus* sp. JF690926.1; *Pseudomonas* sp. JF690929.1; *Exiguobacterium* sp. JF690932.1; *Kocuria* sp. JF690933.1; *Pantoea* sp. JF690934.1; *Pseudomonas* sp. JF690935.1; *Staphylococcus* sp. JF690936.1; *Arthrobacter* sp. JF690937.1; *Comamonas* sp. JF690938.1; *Bacillus* sp. JF690930.1.

### Introduction of bacteria through sugar or blood meals

Bacteria were grown in either LB or ookinete medium overnight at 30 °C, used to seed fresh cultures, and then diluted to an OD_600_ of 1.0, pelleted by centrifugation (10 min, 3000 rpm in a tabletop centrifuge), washed in 1× PBS, and finally resuspended in PBS. For sugar-meal introduction, bacteria were diluted in 3 % sucrose to the concentration indicated in the text and provided to mosquitoes on moistened cotton strips. For blood-meal introduction, mosquitoes were allowed to membrane-feed on blood containing bacteria (10 % bacterial solution, 40 % blood, 50 % human serum) at the concentration indicated in the text. Non-fed mosquitoes were removed within 24 h after blood-feeding.

### *Anopheles gambiae* midgut selection of *Esp_Z*

The parental *Esp_Z* strain was provided to *An. gambiae* mosquitoes via blood meal at 10^9^ CFU/ml (P1). Engorged mosquitoes were maintained under standard insectary conditions, and 10 mosquitoes from this cohort were sampled daily for the presence of *Esp_Z* in the midgut. The *Esp*_Z bacteria that were found in the midgut for the longest time were then used to challenge a second mosquito population, and the process was repeated to isolate the “most fit” *Esp_Z* bacteria for midgut colonization (P2). This process of serial passage was then repeated a third time to isolate the midgut-selected *Esp_Z* bacteria (P3).

### Colonization experiments and DNA extraction

To establish the ability of *Esp_Z* to colonize midguts harbouring a resident microbiota, 70–80 female mosquitoes were treated with antibiotics as described above. Cocktail bacteria (10^6^ CFU/ml) were introduced ad libitum for 48 h via sugar meal; the mosquitoes were then starved for 6–8 h, and *Esp_Z* (10^6^ CFU/ml) was introduced by either blood-feeding or sugar meal. Every 24 h, midguts were dissected from ten individual mosquitoes and transferred to a centrifuge tube containing 100 µl PBS, then stored at −80 °C for DNA extraction; three independent replicates were performed. Mosquitoes were surface-sterilized by washing them in 100 % ethanol for 2 min and then rinsing them for 1 min in sterile 1× PBS. DNA extractions were carried out as previously described [26] with minor modifications: In brief, dissected midguts were homogenized in 90 µl PBS, followed by the addition of 90 µl lysozyme (40 mg/ml) and incubation at 37 °C for 1 h; 300 µl of extraction buffer (1 % SDS; 50 mM Tris–HCl, pH 8.0; 25 mM NaCl; 25 mM EDTA, pH 8.0) was added to samples and incubated at 65 °C for 10 min. Following the addition of 200 µl of 3 M potassium acetate (pH 7.2), samples were incubated on ice for 1 h and then centrifuged at 14,000 rpm in a tabletop centrifuge for 10 min and the supernatants removed. The pellets were then treated with RNase (40 mg/ml) at room temperature for 30 min. The supernatants were extracted twice with phenol/chloroform/isoamyl alcohol and precipitated with 2.5 volumes of 100 % ethanol and 1:10 3 M sodium acetate at −20 °C overnight; the pellets were resuspended in 10 µl of water.

### Absolute qPCR quantification

All mosquito tissues were stored at −80 °C following dissection, and DNA was extracted as described. Absolute qRT-PCR determination was used to determine total bacteria and *Esp_Z* gene copy numbers. Degenerate primers targeting the 16S rRNA sequence of each bacterium were designed from previously sequenced 16S rRNA gene fragments, following Clustal W nucleotide alignment of the sequences [10]. Primers targeting *Esp_Z* gene sequences were designed from *Esp_Z* genomic scaffolds. Primers were checked for potential cross-hybridization through BLASTn searches against the nr gene database, retrieving no significant hits (E value = 0.1). *Esp_Z* primers were tested for specificity for the bacterium through PCR amplification from DNA of *An. gambiae* midguts fed the bacterial cocktail species. PCR fragments used for standard curves were cloned using the pGEM-T Easy vector (Promega). Standard curves for 16S rRNA and *Esp_Z* had efficiencies between 80 and 100 % and R^2^ > 0.99. The relative proportion of *Esp_Z* was calculated by dividing the total number of *Esp_Z*-specific copies by the number of amplified 16S rRNA copies across three biological replicates. Amplification and detection of bacterial DNA was performed using the QuantiTect SYBR Green PCR Kit (Qiagen, Valencia, CA, USA) and ABI Detection System ABI Prism 7000, using two technical replicates. The PCR reaction was performed in a total volume of 20 µl, which consisted of 2× Sybr green master mix (Applied Biosystems, Foster City, California, USA), 75 nM of each primer, and 50 ng of template DNA. The cycling parameters were as follows: initial denaturation at 95 °C for 2 min, followed by 40 cycles of 95 °C for 15 s, 56 °C for 30 s, and 60 °C for 30 s. Following amplification, a melting curve analysis was performed from 60 to 95 °C, collecting fluorescence data every 0.5 °C.

### Relative qPCR quantification

To conduct relative real-time qPCR assays, RNA was extracted using TRIzol^®^ (Life Technologies, Carlsbad, CA) according to the manufacturer’s guidelines and treated with Turbo DNase; first-strand cDNA was produced using Superscript III reverse transcriptase (Invitrogen). cDNA templates were normalized to the *Plasmodium berghei* 18S rRNA gene as previously described [16], and fold changes in gene expression levels were determined using the standard [27]. Assays were performed using Sybr green master mix with the following cycling parameters: initial denaturation at 95 °C for 2 min, followed by 40 cycles of 95 °C for 15 s, 56 °C for 30 s, and 72 °C for 30 s.

### Longevity, fecundity, and fertility assays

To assess the impact of bacterial introduction on mosquito longevity, bacteria were introduced into aseptic female mosquitoes, and the impact of *Esp_Z* was compared to that of the introduction of the bacterial cocktail or a PBS-only control. For each treatment, approximately 60 female mosquitoes were rendered free of their microbiota through antibiotic treatment, and then either *Esp_Z*, the bacterial cocktail, or PBS was introduced by either a blood or sugar meal. Each cohort was then provided with a naïve blood meal at 4 days post-introduction. Non-fed mosquitoes were removed and excluded from the analysis. Mosquito mortality was monitored daily; monitoring continued until all the mosquitoes had perished, and survival percentages were calculated across three biological replicates. Kaplan–Meier survival analysis was carried out using GraphPad Prism 5 software, and *p* values were determined by log-rank test (Mantel-Cox) and corrected for multiple comparisons by the Bonferroni method.

For the fecundity and fertility experiments, 30–60 female mosquitoes were treated with antibiotics as described, and three treatment groups were established with *Esp_Z*, the bacterial cocktail, and PBS being introduced by both sugar and blood meals. Following blood-meal introduction, blood-fed mosquitoes were removed to individual vials (12 ml) lined with moistened filter paper and then incubated under normal rearing conditions. Following sugar-meal introduction, mosquitoes were provided a naïve blood meal 48 h later, and the fed mosquitoes were removed to individual vials. Eggs oviposited on the filter paper were counted daily using a light microscope for 2 days. Females that did not produce eggs by day 2 were re-examined on day 3. After counting, eggs were submerged in water under standard larval rearing conditions, and the hatch rate (fertility) was determined by counting first instar larvae, which were then removed daily. Three biological replicates were performed for the fecundity and fertility assays and significance was determined using the Kruskal–Wallis test.

### *Plasmodium* infection assays

Mosquitoes were fed on NF54 *P. falciparum* gametocyte cultures (0.01 % gametocytaemia; provided by the Johns Hopkins Malaria Institute Parasitology Core Facility) through artificial membranes at 37 °C. The adult mosquitoes were starved for 8–12 h prior to feeding to ensure engorgement, and unfed mosquitoes were removed from the cohort within 24 h. To determine oocyst numbers, the mosquitoes were incubated for a further 7 days at 27 °C, and midguts were dissected out in PBS, stained with 0.2 % mercurochrome, and examined using a light-contrast microscope (Olympus). To establish whether *Esp_Z* is capable of inhibiting *Plasmodium* development when provided via sugar meal, 50–70 female septic mosquitoes were reared as described above and then provided a sugar meal containing *Esp_Z* at varying concentrations for 3–4 days; they were then provided a *P. falciparum*-infected blood meal the following day. Oocyst numbers were determined as described above and compared to a cohort fed only PBS containing 3 % sucrose.

### *Esp_Z* genome sequencing and transcriptome analysis

Total bacterial DNA was extracted from an *Esp_Z* liquid culture. A 3 kb insert paired-end shotgun genomic library was prepared from the extracted DNA and sequenced using the 454 GS FLX Titanium sequencing platform at the Genome Resource Center at the Institute for Genome Sciences (IGS) of the University of Maryland according to the manufacturer’s protocol. Sequencing reads were then assembled using Celera Assembler, and ORFs were predicted and annotated using the IGS Annotation Engine implemented within the CLoVR-Microbe pipeline [28]. Total bacterial RNA from bacterial liquid cultures grown in either LB or ookinete medium was extracted using TRIzol according to the manufacturer’s protocol, treated with Turbo DNase (5U, Life Technologies), cleaned using an RNeasy Mini Kit (Qiagen, Valencia, CA, USA), and the quality determined by an Agilent Bioanalyzer 2100. A custom 8 × 44 K Agilent microarray was designed based on the 454-generated *Esp_Z* genome sequence. Cy-3- or Cy-5-labeled cRNA probes was synthesized from 300 ng of total RNA per replicate using the LabelIT^®^ MicroArray Dual Labeling Kit (Mirus Bio, Madison, WI, USA). Labelled RNA was hybridized overnight at 65 °C, and arrays were scanned with an Agilent scanner. Transcript abundance data were processed and analysed as previously described [29, 30]. In brief, LOWESS normalized background-subtracted median fluorescent values were used to determine Cy5/Cy3 ratios from replicate assays and were then subjected to *t* tests at a significance level of *p* < 0.05 using MIDAS, GEPAS, and TMEV software [31, 32]. The rate for self–self hybridizations was used to calculate a cut-off value for the significance of gene regulation on these microarrays of Log2FC = 0.78 or 1.72-fold regulation [33].

### In vitro parasite culture and co-culture with bacteria

For the rodent malaria parasite production, cultures were carried out as previously described [10] with certain modifications. In brief, *P. berghei* GFP parasites were injected into donor Swiss Webster mice and monitored daily for parasitaemia for 3 days post-infection. Once parasitaemia reached >15 %, infected blood was collected by heart puncture and transferred to phenylhydrazine-treated mice. Mice were treated with pyrimethamine 72 h later and monitored daily for parasitemia and exflagellation. Parasitized mice with ≥20 exflagellation events per 20× microscope field were used for in vitro experiments. Parasitized blood was collected by heart puncture and diluted 1:10 in ookinete medium (10 % foetal bovine serum, 50 μg/ml hypoxanthine, 2 mg/ml NaHCO_3_, 1 μM xanthurenic acid in RPMI 1640 medium, pH 8.3), 4 % RBC lysate, and experiment-specific constituents in a 500 μl total volume. To assess the induction of *P. berghei* antioxidant genes, bacteria (final concentration 10^6^ CFU/ml) were added to the ookinete culture at 0 h. Culture samples were directly transferred to Tri-Reagent (Ambion) at various times, from 1 to 10 h after setup, for total RNA extraction. First-strand cDNA was then prepared for qPCR analysis. Development of ookinetes was confirmed by Giemsa staining at 24 h.

## Results and discussion

### Colonization of the *Anopheles gambiae* midgut by *Esp_Z*

**Fig. 1:**
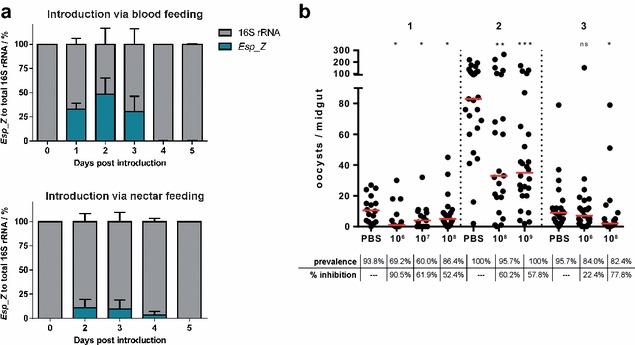
Colonization of the *Anopheles gambiae* midgut by *Esp_Z* and impact on *Plasmodium falciparum* sporogonic development. **a** *Esp_Z* was introduced into a mosquito cohort already containing a cocktail of naturally occurring bacteria through either a blood or sugar meal, and DNA extracted from ten midguts was sampled daily. The *Esp_Z* to 16S rRNA ratio was determined using *Esp_Z*-specific and universal 16S rRNA standard curves. **b** Female *An. gambiae* containing their endogenous microflora were provided with PBS or with *Esp_Z* at the indicated concentration (10× CFU/mL) suspended within 3 % sucrose. After 3 to 4 days of being allowed to feed on these suspensions, they were given a *P. falciparum*-infected blood meal, and oocyst numbers were determined 7 days later. Oocyst counts for three independent replicates are shown (Rep 1–3). *Horizontal bars* represent the median number of oocysts per treatment; inhibition (%) was estimated based on the comparison of these values to that of the PBS control. Prevalence represents the proportion of infected mosquitoes per group. Significance was determined using the Mann–Whitney test by comparing treatment groups to their respective control cohorts (*p < 0.05; **p < 0.01; ***p < 0.001)

*Esp_Z* has been detected within the midguts of field-caught anopheline malaria vectors [10], but its capacity to persist within the mosquito following artificial introduction is not known. The extent to which *Esp_Z* can colonize the mosquito midgut, the site of *Plasmodium* pre-oocyst development, is likely to contribute to the magnitude of the *Plasmodium* inhibition. The persistence of *Esp_Z* in the mosquito midgut when co-present with a simulated natural microbiota was determined. The midgut microflora of field-caught mosquitoes has been shown to differ markedly from its laboratory-reared counterparts [7, 34]; therefore, a cocktail of bacteria previously isolated from the same southern Zambian populations of *An. arabiensis* from which *Esp_Z* was identified was utilized [10]. To determine the relative abundance of *Esp_Z* over time, the ratio of *Esp_Z* to total bacterial 16S rRNA was calculated through quantitative real-time PCR. Two approaches were taken to introduce *Esp_Z* into mosquitoes already harbouring a resident microbiota: either through a blood meal as shown before for this bacterium [10] or through a sugar meal to inquire on the viability of introducing *Esp_Z* to anophelines via nectar feeding. *Esp_Z* bacteria were able to persist within the midguts of cocktail-colonized mosquitoes following blood-meal introduction for up to 72 h. *Esp_Z* constituted an average 32.8, 48.5, and 30.3 % of the total bacterial population at 24, 48, and 72 h post-introduction, respectively. *Esp_Z* bacteria were detected up to 120 h post-introduction but reflected <1 % of the total microbiota (Fig. 1a, top). Following the introduction of *Esp_Z* through sugar feeding into mosquitoes already harbouring a cocktail microbiota, *Esp_Z* bacteria were detected for 4 days, peaking at an initial 11.1 % of the total microbiota following introduction and still detectable (3.5 %) at day 4 (Fig. 1a, bottom).

Although the bacterial cocktail consisted of strains isolated from field mosquitoes, the microflora of individual mosquitoes is highly variable [7]. Therefore, the persistence of *Esp_Z* for 4 days in the presence of natural microflora is encouraging. It also indicates that, when possible, a bacterium destined for field-based release must be investigated in the context of a natural microbiota. In addition, acquisition of a sugar meal provides energy reserves for flight [35] and promotes mosquito longevity [25, 36], suggesting that multiple sugar meals may be taken during the mosquitoes’ lifespan. Note that when introduced through the sugar meal, *Esp_Z* tended to constitute a lower proportion of the total microbiota when compared to ingestion by blood-feeding (Fig. 1a). In the field, it is estimated that *Anopheles* mosquitoes feed on blood every two or 3 days [37, 38]; knowing there is a rapid expansion of the gut microbiota upon blood intake [39, 40], it is reasonable to assume that *Esp_Z*, even when introduced by nectar feeding, could persist in the mosquito gut beyond what was observed in a laboratory setting. Therefore, when considering a field-based application of bacteria, sugar-meal introduction would be best evaluated in the context of the specificities of the anophelines and their feeding behaviour in any given ecological niche. In summary, it is shown that *Esp_Z* can be introduced through both bacteria-enriched blood and sugar sources and persist for multiple days in competition with a natural microbiota.

### Inhibition of *Plasmodium* sporogonic development following sugar-meal introduction of *Esp_Z*

Having determined that *Esp_Z* can persist within the mosquito midgut for up to 4 days following sugar-meal introduction, the ability of *Esp_Z* to inhibit *Plasmodium* development when provided to mosquito cohorts through a sugar meal was investigated next. Previous experiments have shown *Esp_Z* is highly effective at inhibiting *Plasmodium* development at the ookinete stage when provided in a blood meal [10]. Use of attractive toxic sugar baits has proven successful in reducing mosquito populations [41], indicating that bacteria, in this context, could be disseminated through sugar feeding. To simulate an approximation of how *Esp_Z* bacteria would be disseminated through a natural population, mosquitoes, containing their endogenous microflora, were provided *Esp_Z* suspended in 3 % sucrose and allowed to feed for three to 4 days. *Plasmodium* infection assays revealed that the impact of *Esp_Z* upon parasite development when provided through a sugar meal averaged a 45–65 % inhibition across the different concentrations tested (Fig. 1b). Of note, however, is the inter-replicate variability that was observed, especially at the lowest tested concentration of 10^6^ CFU/mL; inhibition varied from 22.2 % in one replicate (from May 2014) to 90.5 % in another (February 2014). Seasonal variations in the mosquitoes’ resident microbiota may provide an explanation for these results. In particular, the midguts of mosquitoes fed in May 2014 were dominated by a different bacterium as observed by the colonies that resulted of plating ground midguts in LB agar. It is also likely that individual mosquitoes actually fed on the *Esp_Z* sucrose solution at different times prior to the infected blood meal, suggesting that they would harbour different *Esp_Z* concentrations and explaining how these results might differ from the ones obtained when *Esp_Z* was introduced directly with the blood meal [10]. Within 24 h after mosquito ingestion of *Plasmodium* gametocytes, the number of developing ookinetes is substantially lower than the initial gametocyte number [42], and this bottleneck occurs as the result of numerous factors [5]. Therefore, if detectable numbers of *Esp_Z* are present 24 h after an infectious blood meal, they are likely to further constrict this bottleneck and reduce *Plasmodium* transmission.

### *Esp_Z* influence on mosquito fitness parameters

**Fig. 2:**
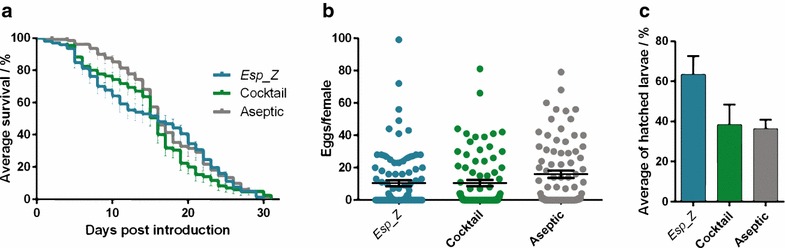
Sugar-meal introduction of *Esp_Z* has no impact on *Anopheles gambiae* fitness. **a** Longevity studies were performed following continuous sugar-meal introduction of either *Esp_Z*, a bacterial cocktail, or PBS into aseptic mosquito cohorts. A sterile blood meal was provided on day 4 (*black arrow*), and unfed mosquitoes were censored from the analysis. Survival was monitored daily and continued until 100 % mortality was reached. The *curves* represent the average percent mortality across three replicates; the *error bars* indicate standard error. Significance was determined using the log-rank test (Mantel-Cox) with Bonferroni correction using a Kaplan–Meier survival analysis. *Esp_Z* vs Cocktail, *p* > 0.9999; *Esp_Z* vs Aseptic, *p* = 0.3852. **b** Comparative fecundity analysis of the *Esp_Z*-, bacterial cocktail- and PBS-fed, sugar-fed mosquito cohorts. Separate cohorts were provided with a blood meal 72 h after introduction, and *circles* represent the number of eggs laid per female. *Horizontal bars* represent the median number of eggs, and *error bars* indicate the standard error; three pooled biological replicates are shown. *p* = 0.0596 (Kruskal–Wallis test). **c** Fertility analysis in *Esp_Z*-, bacterial cocktail-, and PBS-fed mosquitoes. At 72 h after introduction, mosquitoes were offered a blood meal, and those not engorged were removed. Eggs were collected at 48 h post-blood meal and allowed to hatch in rearing trays. The hatch rate indicates the percentage of eggs giving rise to 1st instar larvae; the *error bars* indicate the standard error of the mean. *p* = 0.0741 (Kruskal–Wallis test)

It has previously been shown that the reintroduction of naturally occurring bacteria can affect mosquito fitness [9]. Recently, a *Chromobacterium* species has been identified that displays both anti-parasitic and entomopathogenic properties and may therefore be used to reduce both disease transmission and vector populations [15]. To provide insight into the impact of *Esp_Z* bacteria on *An. gambiae* physiology, its effects on mosquito longevity, fecundity, and fertility were assayed following introduction via the two methods, a blood meal and continuous sugar feeding. To assay longevity, *Esp_Z*, the bacterial cocktail, or a bacteria-free PBS control were introduced into aseptic mosquitoes and monitored mortality daily. Following introduction via sugar-feeding, there was no significant difference between the mosquitoes harbouring no bacteria (aseptic) and those continuously fed *Esp_Z* (*p* value = 0.3852), or those harbouring the bacterial cocktail and those fed to *Esp_Z* (*p* > 0.9999), all having a median survival of 16 days (Fig. 2a). Conversely, when introduced through the blood meal, *Esp_Z* had a significant impact on longevity when compared to the bacterial cocktail (*p* = 0.0004) and to the aseptic group (*p* = 0.0308) (Additional file 1: Figure S1A). The presence of *Esp_Z,* introduced by either method, did not significantly affect egg production (via sugar meal, Fig. 2b, *p* = 0.0596; via blood meal, Additional file 1: Figure S1B, *p* = 0.0507) nor mosquito fertility measured by hatched larvae (via sugar meal, Fig. 2c, *p* = 0.0741; via blood meal, Additional file 1: Figure S1C, *p* = 0.1000).

There was no impact of *Esp_Z* on mosquito survival when provided in a sugar meal (Fig. 2a), whereas blood-feeding significantly negatively affected mosquito longevity (Additional file 1: Figure S1). This result is perhaps expected, since acquisition of the native microflora likely occurs through sugar meals [39, 40], whereas blood-meal introduction is an artificial system and would result in a sudden expansion of the midgut microbiota. Taken together, these data suggest that in a field-based approach, *Esp_Z* introduced through sugar-baited traps would not adversely affect mosquito fitness and, therefore, the presence of *Esp_Z* would not be selected against when artificially reintroduced into field mosquitoes.

### *Esp_Z* genome analysis

**Fig. 3:**
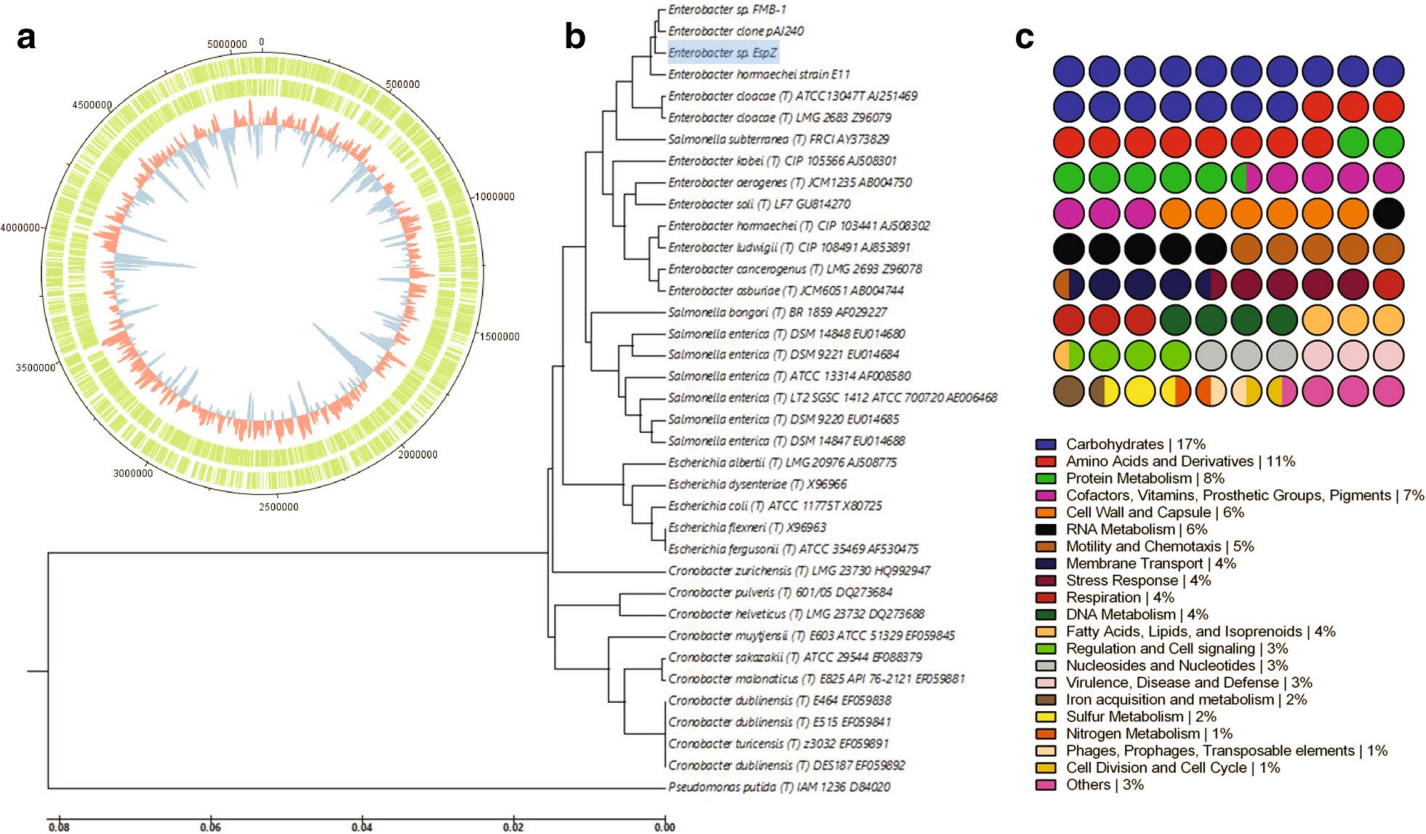
*Esp_Z* genome. **a** Circular representation of the *Esp_Z* genome. The coding regions of both the forward and reverse strands are shown in *green*; *blue* represents a negative deviation and *red* a positive deviation of the average G–C content for this genome of 55.8 %. **b** Evolutionary relationships between *Esp_Z* and related type and non-type strains, based on 16S rRNA genomic sequences and inferred by the UPGMA method. The optimal tree with the sum of branch lengths equal to 0.33591652 is shown. The tree is drawn to scale, with branch lengths in the same units as those of the evolutionary distances used to infer the phylogenetic tree. The evolutionary distances were computed using the maximum composite likelihood method and are expressed as the number of base substitutions per site. The analysis was conducted in MEGA6 and involved 37 genomic sequences. **c** Distribution of the functional role categories of the protein-encoding genes of *Esp_Z* using the SEED database; 47 % of those genes were not assigned a functional role and are omitted from this representation

To gain insight into the genetic basis of the *Esp_Z*-mediated inhibition of *Plasmodium* and develop tools for bacterial functional genomics, the *Esp_Z* genome was sequenced via a whole-genome shotgun sequencing approach, as described in “Methods” section. A total of 864,207 Titanium sequencing reads were obtained, corresponding to an approximately 38× genome coverage. After assembly and ORF prediction, a total of 39 scaffolds were obtained, two of which were identified as plasmid-related through identity with known plasmid sequences in GenBank. PCR and Sanger-based sequencing analysis confirmed the presence of two closed plasmids. The estimated size of the chromosomal *Esp_Z* genome is then 5.2 Mbp, with a GC-content averaging 55.8 % (Fig. 3a). A phylogenetic analysis based on 16S rRNA genomic sequences allowed for the confirmation of *Esp_Z* as a novel *Enterobacter* species (Fig. 3b). The *Esp_Z* genome contains 4919 putative protein-coding genes that were subjected to SEED functional analysis (Fig. 3c) and used to construct microarrays for whole-genome expression analysis.

### Transcriptomic survey of *Esp_Z* genes that mediate selection in the mosquito midgut environment

**Fig. 4:**
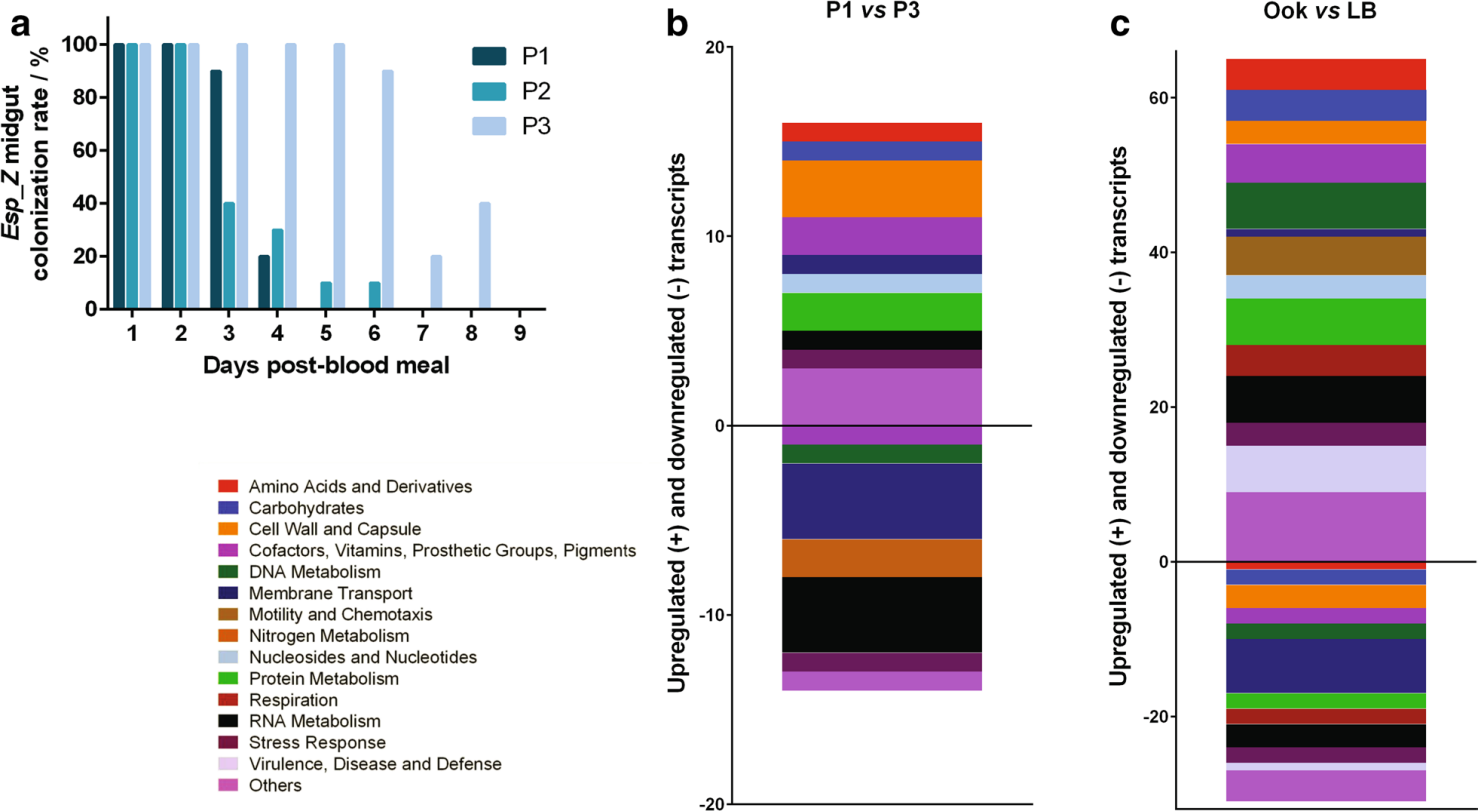
Differential gene expression in *Esp_Z* on different culture media and upon serial passage in the *Anopheles gambiae* midgut. **a** A parental strain of *Esp_Z* (P1) was provided to *An. gambiae* mosquitoes through a blood meal and sampled daily. Colonies persisting for the longest time were reintroduced into a second cohort (P2) and the process repeated a third time to isolate the passaged (P3) strain. The ability of a selected strain of *Esp_Z* to persist within the *An. gambiae* midgut was determined through qRT-PCR copy number analysis. **b** Distribution of the functional role category of upregulated (+) and downregulated (−) transcripts of parental P1 when compared to passaged P3. Functional role categories were assigned to 59/98 upregulated and 29/61 downregulated transcripts using the SEED database. **c** Distribution of the functional role category of upregulated (+) and downregulated (−) transcripts of *Esp_Z* grown in ookinete medium when compared to LB medium. Functional role categories were assigned to 15/21 upregulated and 14/20 downregulated transcripts using the SEED database

To enhance the process of *Anopheles* midgut colonization, the *Esp_Z* bacterium was serially passaged and selected for prolonged persistence in the mosquito midgut, as described previously [43]. The persistence of the parental *Esp_Z* strain was able to increase from four to eight days by selecting for bacteria best able to survive in the mosquito gut (Fig. 4a). A total of 41 genes were differentially regulated between the parental (P1) and passaged (P3) *Esp_Z* strains, 20 genes with increased expression in the passaged strain and 21 in the parental (Fig. 4b; Additional file 2: Table S1).

Of particular interest within the 20 regulated genes of the passaged bacterium were a type III secretion apparatus protein, a sugar transport component, a glutathione S-transferase, and an oxidoreductase (Table 1). Type III secretion systems have been shown to modulate eukaryotic host immune cell signalling, allowing colonization of the host [44, 45]. *Esp_Z*_3940 (Log2FC = 1.07) showed the highest similarity to *Pantoea* sp. Type III secretion system export protein SpaR/YscT/HrcT, and a *Pantoea* Type III secretion has been shown to be important for bacterial persistence in an insect vector, the flea beetle *Chaetocnema pulicaria* [46]. A sugar ABC transporter component was also found in increased abundance in the passaged strain (Log2FC = 0.83). A component of the sugar transport system has previously been identified as a key virulence factor in bacterial colonization [47], and during *Bacillus cereus* colonization of the honeycomb moth, a sugar sensing and transport operon is upregulated [48]. Because sugars and glucosidases are present in the mosquito midgut, it is possible that *Esp_Z* adapts to its host environment through increased expression of sugar transport genes to allow the utilization of available nutrients. Following ingestion of a blood meal, the mosquito midgut environment is rich in haem, which can modulate the oxidative state of the midgut environment [49]. An increased abundance of GST (Log2FC = 0.88) and oxidoreductase (Log2FC = 0.84) transcripts in the selected bacterium was observed, suggesting that the passaged strain is able to elevate its response to the harsh ROS rich environment in order to maintain redox homeostasis and persist within the mosquito midgut. Sugar-fed *Aedes aegypti* mosquitoes constitutively express ROS in the midgut, so it is possible that the selected bacterium elevates antioxidant genes in both the sugar- and blood-fed environments [50].

**Table 1:** Colonization adaptation-related candidate genes that are differentially regulated between the parental (P1) and adapted (P3) *Esp_Z* strains

Gene ID	Description	Process GO category	Fold change (log_2_)
*Parental upregulated*			
ESP_Z_1310	yadA-like *C*-terminal region family protein	N/A	0.999
ESP_Z_1067	SOS cell division inhibitor	SOS response	0.897
ESP_Z_3679	uxuR transcriptional repressor	Regulation of transcription, DNA-dependent	0.834
*Adapted upregulated*			
ESP_Z_2042	Type III secretion apparatus protein	Transport	1.065
ESP_Z_4047	Glutathione S-transferase, *C*-terminal domain protein	Metabolic process	0.878
ESP_Z_0990	Oxidoreductase FAD-binding domain protein	Oxidation–reduction process	0.836
ESP_Z_4206	Sugar abc transporter permease	Transport	0.827

Amongst the genes with increased expression in the parental *Esp_Z* strain (Table 1), YadA transcripts (Log2FC = 1.0) are of particular note. YadA is a bacterial adhesin able to mediate binding to epithelial cells [51, 52] and may putatively align *Esp_Z* in close proximity to the mosquito midgut epithelium and result in elicitation of immune response [53]. Decreased YadA transcription in the passaged strain may therefore have the potential to prevent co-localization with the midgut epithelium and allow colonization without detrimental stimulation of an antibacterial immune response. UxuR, also found to have increased abundance in the parental strain (Log2FC = 0.83), is a transcription factor that negatively regulates the expression of UidA, a key enzyme of the hexuronate metabolic pathway in *Escherichia coli* [54, 55]. Decreased expression of *uxuR* in the passaged strain is therefore in line with the previous hypothesis that sugar transport may be involved in the selection process: a lower abundance of the *uxuR* transcriptional repressor would allow greater utilization of sugar in the mosquito midgut as a nutrient source. *sulA* (Log2FC = 0.90) is regulated by the SOS response, an inducible DNA repair mechanism, and halts cell division to prevent proliferation of DNA damage. The bacterial SOS response can be activated by exposure to antimicrobials and changes in oxidative conditions [56], conditions that prevail in the mosquito midgut. This hostile environment may cause upregulation of *sulA* in the parental strain and, conversely, lower expression in the passaged strain, since it is tolerant to the environment.

This microarray analysis has revealed that elevated expression of genes involved in response to the external environment likely contributes to the ability of the passaged *Esp_Z* strain to sense the local environment, and it is critical to the bacterium’s ability to more effectively colonize the mosquito midgut. In addition, decreases in the abundance of transcripts involved in the SOS damage response and repression of sugar metabolism suggest that the selected strain is more suited to the hostile midgut environment.

### Transcriptomic survey for *Esp_Z*-encoded *Plasmodium* inhibition factors

Previous experiments herein have shown that *Esp_Z* inhibits *Plasmodium* ookinete formation via a mechanism that involves bacterial production of an oxidative environment. This mechanism is likely controlled either directly or indirectly by a genetic component of the bacteria. Earlier studies also showed that *Esp_Z* produces factors that inhibit parasite development in vitro when cultured in an ookinete medium, but not when cultured under conditions of basic replication in LB broth [10]. To identify putative *Esp_Z* genes involved in the ROS-mediated killing of *Plasmodium* parasites, the same whole-genome microarray approach to compare gene expression between *Esp_Z* grown in ookinete and LB media was employed, since only the ookinete medium induces ROS production [10]. A total of 98 and 61 genes enriched in ookinete medium- and LB medium-grown *Esp_Z* were identified, respectively (Fig. 4c; Additional file 2: Table S1). Sequences within the gene ontology groups that included oxidation–reduction process, transport, and respiratory electron transport chain were identified.

Genes of particular interest with increased abundance under ookinete medium conditions are summarized in Table 2. Upregulation of a transcript containing molybdenum-pterin binding domains was observed (Log2FC = 1.17) and proteins with such domains are important co-factors for the acquisition of molybdenum. This trace element functions as a catalyst for molybdenum-dependent two-electron reduction–oxidation (redox) reactions [57], indicating that upregulation of this transcript in the ookinete medium-cultured bacteria may lead to the production of elevated ROS levels. GTP cyclohydrolase II (Log2FC = 1.38) catalyses the first step of the biosynthesis pathway for riboflavin [58], an important precursor to bacterial redox sensors such as flavin mononucleotide (FMN) and flavin adenine dinucleotide (FAD) [59]. Elevated expression of this redox sensor precursor may suggest that ookinete medium-derived *Esp_Z* bacteria are responding to the presence of elevated ROS levels. We also observed increased abundance of a putative NADPH nitroreductase (Log2FC = 1.36). Nitroreductases donate electrons to nitrogen-containing aromatic compounds generating ROS in the process [60], and this process has been suggested to mediate the trypanocidal activity of Nifurtimox and Benznidazole [61]. Interestingly, nitroreductases require either FMN or FAD as a co-factor for the generation of free radicals [62]: upregulation of both an NADPH nitroreductase and GTP cyclohydrolase II suggests that ookinete medium-cultured *Esp_Z* are generating ROS at least partially through the reduction of flavins.

**Table 2:** ROS-producing candidate genes that are differentially regulated between *Esp_Z* grown in ookinete medium conducive to ROS production, and LB medium

Gene ID	Description	Process GO category	Fold change (log_2_)
ESP_Z_1948	GTP cyclohydrolase II	Riboflavin biosynthetic process	1.384
ESP_Z_1388	NADPH nitroreductase	Oxidation–reduction process	1.359
ESP_Z_4666	Molybdenum-pterin binding domain protein	Transmembrane transport	1.169
ESP_Z_1849	Cytochrome b561 family protein	Respiratory electron transport chain	1.105

In addition, a cytochrome b561, upregulated in the ookinete medium-grown *Esp_Z* (Log2FC = 1.11), has been shown to allow for regeneration of the reduced, ROS-detoxifying form of ascorbic acid [63]. It has previously been shown that supplementation with ascorbic acid or reduced glutathione and the resulting decreased redox environment limit the ability of *Esp_Z* to inhibit *Plasmodium* development [10]. Upregulation of a reducer of ascorbic acid suggests that the ookinete medium-derived *Esp_Z* bacteria are in an increased ROS environment and require an increased abundance of ascorbic acid in its reduced, active state. It becomes apparent that *Esp_Z*, when grown in an environment conducive to ROS production, elevates transcripts associated with the generation of free radicals, and a combination of pathways are likely to contribute to the generation of anti-*Plasmodium* reactive species.

### Influence of *Esp_Z* on *Plasmodium* antioxidant gene expression

We have previously shown that *Esp_Z* mediates *Plasmodium* killing through a ROS-dependent mechanism, as supplementation with antioxidants, such as ascorbic acid or reduced glutathione, limits the ability of *Esp_Z* to inhibit *Plasmodium* development [10]. In addition, since genes conducive to an elevated ROS environment are upregulated within this bacterium (Table 2), it lead to the hypothesis that the production of ROS may affect the expression of key *Plasmodium* antioxidant genes. To test this possibility, in vitro ookinete cultures of *P. berghei* were exposed to *Esp_Z*, a control anti-*Plasmodium* bacteria that does not produce ROS (*Pseudomonas putida, Ppu*) [10], or to a PBS control, and the expression of five parasite antioxidant genes was measured over time: thioredoxin reductase (*trxr)*, thioredoxin (*trx)*, thioredoxin 1 (*trx1)*, peroxiredoxin-1 (*tpx*-*1)*, and 1-Cys peroxiredoxin (*1*-*cys*-*prx)*. Expression of *tpx*-*1* and *1*-*cys prx* have previously been shown to peak at 12 and 24 h, respectively, after initiation of the ookinetes culture, and both increase in abundance in the presence of a superoxide producer; no impact on *trx* expression was detected [16]. *Plasmodium bergheitpx*-*1* knockout lines have been shown to have reduced gametocyte numbers [64] and to exhibit increased numbers of immature oocysts and, consequently, a reduction in the number of sporozoites [65].

**Fig. 5:**
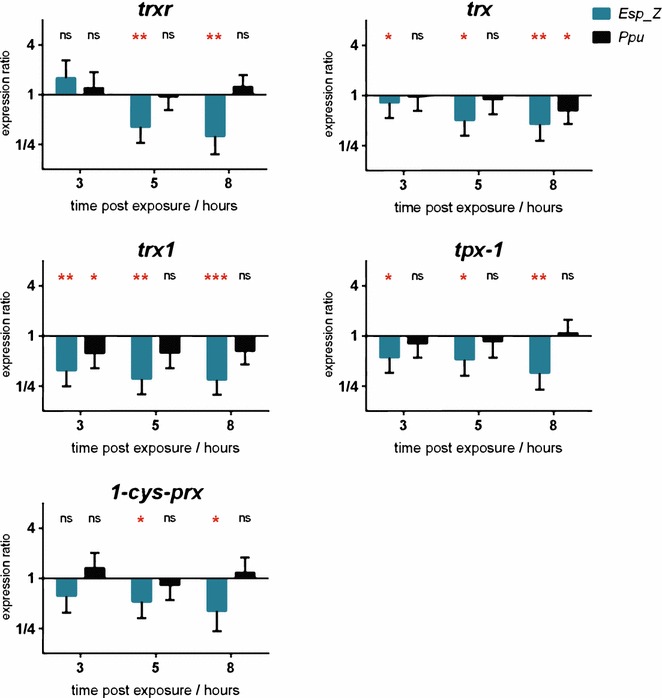
Exposure to *Esp_Z* limits *Plasmodium*’s antioxidant response. Relative *P. berghei* pre-ookinete expression of thioredoxin reductase (*trxr)*, thioredoxin (*trx)*, thioredoxin 1 (*trx1)*, peroxiredoxin-1 (*tpx*-*1)*, and 1-Cys peroxiredoxin (*1*-*cys*-*prx*) upon in vitro co-culture with *Esp_Z* (*blue*) or *Pseudomonas putida* (*Ppu*, black). RT-qPCR datasets were normalized to the level of expression of 18SrRNA, a PBS control, and the expression at 1 h post-exposure for each experimental group. *Bars* represent the mean ± the standard error of the mean of six biological replicates over two independent experiments (3 + 3). Significance was determined using a two-tailed one sample t-test to determine whether each transcript was significantly* up*- or* down*-regulated (*p < 0.05; **p < 0.01; ***p < 0.001)

Pooling the results of two independent experiments (each including three biological replicates), we observed a reduction in transcript abundance of all targets 8 h after treatment with *Esp_Z* (Fig. 5). It is important to understand that arrested parasite development becomes apparent shortly after 8 h of co-culture with *Esp_Z* [10]. For all genes, the transcript abundance did not alter significantly when the ookinete cultures were exposed to *Pseudomonas putida*, nor were there significant differences on antioxidant gene expression between exposure to the ROS-producing *Esp_Z* or this non-ROS inducing *Pseudomonas putida*. Of note, also, is the fact that the expression of 1-Cys-peroxiredoxin seemed to follow a trend similar to that of the more canonical thioredoxin system enzymes, even though it has been suggested that in *P. berghei1*-*cys prx* acts primarily to protect against heme-derived endogenous ROS, rather than exogenous ROS [66]. Taken together, these data indicate the possibility that *Esp_Z* interferes with ookinete maturation by partially and temporarily inducing a shutdown of the parasite’s antioxidant response.

*Plasmodium falciparum tpx*-*1* knockout lines display an increased sensitivity to the presence of superoxide producers [22], and expression of *P. berghei tpx*-*1* and *1*-*cysprx* is increased in response to external ROS [16]. These results suggest that the downregulation of thioredoxin-1 and the thioredoxin-dependent peroxiredoxins by *Esp_Z* renders the parasite more susceptible to the presence of *Esp_Z*-derived ROS [10]. *P. bergheitpx*-*1* knockout lines demonstrate a reduced sporozoite load when compared to wild-type parasites [65], suggesting that the anti-parasite phenotype of *Esp_Z* may, in part, be due to a negative effect on the parasite’s antioxidant defences.

## Conclusions

The devastating effects of malaria transmission are ever-present in sub-Saharan Africa, and increasing efforts to counteract these effects are either encountering resistance, as in the case of anti-malarial drugs and insecticides, or poor efficiencies, in the case of vaccine development. It is, therefore, imperative that alternate strategies be explored, and the microbiota of disease vectors has become an attractive resource for this purpose. Not only do certain members of the vector microbiota nearly eliminate *Plasmodium* infection [9, 10], but some members have also been shown to effectively kill the mosquito vectors of several pathogens [15]. Here, the potential of the naturally occurring *Enterobacter* species *Esp_Z* as part of a malaria control strategy was evaluated.

*Proteobacteria* are highly abundant in field-caught mosquitoes, including *Enterobacter* species, showing that they can be acquired and persist in the mosquito and its habitat [7, 8]. In order to influence the natural microbiota, bacteria could be introduced via sugar-baited traps [41] thus ensuring *Plasmodium* exposure to them. It was demonstrated that *Esp_Z* can persist for up to four days after introduction and inhibit *P. falciparum* development when introduced via sugar meal (Fig. 1). As part of a translation study, it is important to examine the introduction and retention of bacteria in the adult midgut, because of the as-yet unclear data on transmission of bacteria from larvae to adults [67–69].

The impact on mosquito fitness of an introduced bacterium is an additional consideration, since a potentially negative impact is likely to be selected against in nature. Unlike the intracellular *Wolbachia* species, bacteria that are able to manipulate the host’s reproductive system and thereby ensure vertical dissemination through the population [70, 71], the spread of a bacterium must not induce any fitness cost. Microbiota are important for mosquito fitness, with the proteobacteria *Asaia* sp. influencing larval development and several other species having been shown to affect mosquito fitness [9, 72]. Naturally occurring bacterial isolates have a varying ability to persist and replicate when re-introduced into aseptic *An. gambiae* midguts [9], suggesting that there is variation in the adaptation to the mosquito midgut environment. This study has shed light on candidate molecular components that are important for bacterial colonization and persistence in the mosquito. It is possible that a combinatorial approach could be undertaken, utilizing a cocktail of anti-parasitic bacteria, each having a different anti-parasitic mechanism, for the control of malaria transmission.

In the present study, the nature of the anti-*Plasmodium* activity of *Esp_Z* was also further characterized. On the one hand, the observation that *Esp_Z* upregulates genes associated with the generation of ROS when cultured under conditions shown to inhibit parasite development in vitro was validated. This finding corroborates previous studies in which *Esp_Z* was shown to induce an oxidative environment when co-cultured with *Plasmodium* ookinetes, with such an environment being the likely cause of parasite inhibition. On the other hand, a potential complementary mechanism by which the bacteria appears to directly interfere with the parasite’s antioxidant system was uncovered, thereby limiting its ability to respond and adapt to the oxidative environment it promotes. This mechanism is indicated by the downregulation of key genes of the thioredoxin and glutathione antioxidant pathways in *P. berghei* ookinetes, until the moment when parasite development is arrested. Current efforts are underway to identify putative mediators of this effect in order to further characterize this phenomenon, in what also constitutes an exploration of *Esp_Z*’s metabolome from a natural product discovery perspective, in the search for new and innovative anti-malarials.

## Additional files

10.1186/s12936-016-1468-2 Impact of blood feeding *Esp_Z* on mosquito fitness. (**A**) Longevity studies were performed following blood-meal introduction of either *Esp_Z*, bacterial cocktail, or PBS into aseptic mosquito cohorts. A sterile blood meal was provided on day 4 (black arrow), and unfed mosquitoes were censored from the analysis. Survival was monitored daily and continued until 100 % mortality was reached. The curves represent the average percent mortality across three replicates, and the error bars indicate the standard error. Significance was determined using the log-rank test (Mantel-Cox) using a Kaplan–Meier survival analysis. (**B**) Fecundity analysis between *Esp_Z*-, bacterial cocktail- and PBS-fed blood-fed mosquito cohorts. Separate cohorts were provided a second blood meal 72 h after blood-meal introduction of *Esp_Z*, and circles represent the number of eggs laid per female. Horizontal bars represent the median number of eggs, error bars indicate the standard error, and three pooled biological replicates are shown. Significance was determined using the Mann–Whitney test. (**C**) Fertility analysis in *Esp_Z*-, bacterial cocktail-, and PBS-fed blood-fed mosquitoes. At 72 h after introduction, mosquitoes were offered a second, naive blood meal, and those not engorged were removed. Eggs were collected 48 h post-blood meal and allowed to hatch in rearing trays. The hatch rate indicates the percentage of eggs giving rise to 1st instar larvae; the error bars indicate the standard error of the mean, and significance was determined using an unpaired *t*-test
10.1186/s12936-016-1468-2 Global results of DNA microarray analysis of *Esp_Z* transcriptome upon selection in the mosquito midgut environment (P1 vs. P3) and upon growth in *Plasmodium* inhibiting conditions (Ook vs. LB).

## Abbreviations

ROS: reactive oxygen species
CFU: colony forming units
*Esp_Z*: *Enterobacter* sp. Zambiae
IGS: Institute for Genome Sciences
Log2FC: binary logarithm of fold change
redox: reduction–oxidation
FMN: flavin mononucleotide
FAD: flavin adenine dinucleotide
Tpx-1: peroxiredoxin-1
1-Cys-prx: 1-cys-peroxiredoxin
Trx-1: thioredoxin-1
*trxr*: thioredoxin reductase
*trx*: thioredoxin
*trx1*: thioredoxin 1
*tpx*-*1*: peroxiredoxin-1
*1*-*cys*-*prx*: 1-Cys peroxiredoxin
TPx_Gl_: peroxidase-like thioredoxin peroxidase
