# Supplementary material for: Functional genomic analyses of Enterobacter, Anopheles and Plasmodium reciprocal interactions that impact vector competence
Source: Malar J. 2016 Aug 22;15(1):425. doi: 10.1186/s12936-016-1468-2 (PMC4994321; doi:10.1186/s12936-016-1468-2)
Supplement: Supplementary file 1 — 10.1186/s12936-016-1468-2 Impact of blood feeding Esp_Z on mosquito fitness. (A) Longevity studies were performed following blood-meal introduction of either Esp_Z, bacterial cocktail, or PBS into aseptic mosquito cohorts. A sterile blood meal was provided on day 4 (black arrow), and unfed mosquitoes were censored from the analysis. Survival was monitored daily and continued until 100 % mortality was reached. The curves represent the average percent mortality across three replicates, and the error bars indicate the standard error. Significance was determined using the log-rank test (Mantel-Cox) using a Kaplan–Meier survival analysis. (B) Fecundity analysis between Esp_Z-, bacterial cocktail- and PBS-fed blood-fed mosquito cohorts. Separate cohorts were provided a second blood meal 72 h after blood-meal introduction of Esp_Z, and circles represent the number of eggs laid per female. Horizontal bars represent the median number of eggs, error bars indicate the standard error, and three pooled biological replicates are shown. Significance was determined using the Mann–Whitney test. (C) Fertility analysis in Esp_Z-, bacterial cocktail-, and PBS-fed blood-fed mosquitoes. At 72 h after introduction, mosquitoes were offered a second, naive blood meal, and those not engorged were removed. Eggs were collected 48 h post-blood meal and allowed to hatch in rearing trays. The hatch rate indicates the percentage of eggs giving rise to 1st instar larvae; the error bars indicate the standard error of the mean, and significance was determined using an unpaired t-test [file 12936_2016_1468_MOESM1_ESM.docx]

**SUPPLEMENTARY MATERIAL**

**
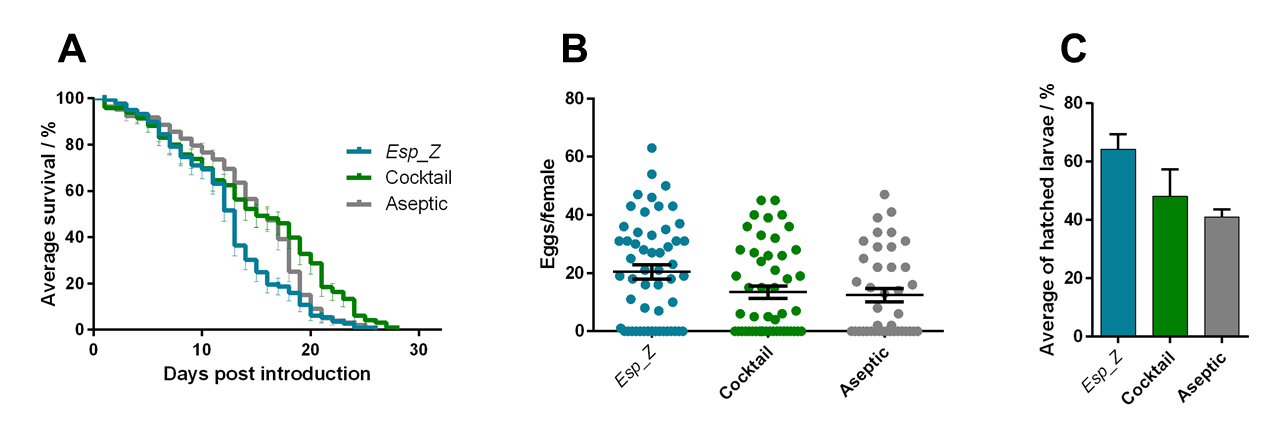
**

**Additional file 1: Figure S1: Impact of blood feeding *Esp_Z* on mosquito fitness**

(**A**) Longevity studies were performed following blood-meal introduction of either *Esp_Z*, bacterial cocktail, or PBS into aseptic mosquito cohorts. A sterile blood meal was provided on day 4 (black arrow), and unfed mosquitoes were censored from the analysis. Survival was monitored daily and continued until 100% mortality was reached. The curves represent the average percent mortality across three replicates, and the error bars indicate the standard error. Significance was determined using the log-rank test (Mantel-Cox) using a Kaplan-Meier survival analysis. (**B**) Fecundity analysis between *Esp_Z-*, bacterial cocktail- and PBS-fed blood-fed mosquito cohorts. Separate cohorts were provided a second blood meal 72 h after blood-meal introduction of *Esp_Z*, and circles represent the number of eggs laid per female. Horizontal bars represent the median number of eggs, error bars indicate the standard error, and three pooled biological replicates are shown. Significance was determined using the Mann-Whitney test. (**C**) Fertility analysis in *Esp_Z-*, bacterial cocktail-, and PBS-fed blood-fed mosquitoes. At 72 h after introduction, mosquitoes were offered a second, naive blood meal, and those not engorged were removed. Eggs were collected 48 h post-blood meal and allowed to hatch in rearing trays. The hatch rate indicates the percentage of eggs giving rise to 1^st^ instar larvae; the error bars indicate the standard error of the mean, and significance was determined using an unpaired *t*-test.
